# Epigenome-wide association study of global cortical volumes in generation Scotland: Scottish family health study

**DOI:** 10.1080/15592294.2021.1997404

**Published:** 2021-11-23

**Authors:** Miruna Carmen Barbu, Mat Harris, Xueyi Shen, Stolicyn Aleks, Claire Green, Carmen Amador, Rosie Walker, Stewart Morris, Mark Adams, Anca Sandu, Christopher McNeil, Gordon Waiter, Kathryn Evans, Archie Campbell, Joanna Wardlaw, Douglas Steele, Alison Murray, David Porteous, Andrew McIntosh, Heather Whalley

**Affiliations:** a Division of Psychiatry, The University of Edinburgh, Royal Edinburgh Hospital, Edinburgh, UK; b Mrc Human Genetics Unit, Institute of Genetics and Cancer, the University of Edinburgh, UK; c Centre for Genomic and Experimental Medicine, Institute of Genetics and Cancer, the University of Edinburgh, UK; d Centre for Clinical Brain Sciences, The University of Edinburgh, UK; e Aberdeen Biomedical Imaging Centre, The Institute of Medical Sciences, University of Aberdeen, UK; f Imaging Science and Technology, School of Medicine, University of Dundee, Dundee UK; g Centre for Cognitive Ageing and Cognitive Epidemiology, School of Philosophy, Psychology and Language Sciences, The University of Edinburgh, UK

**Keywords:** DNA methylation, epigenome-wide association study, cortical volumes, generation Scotland

## Abstract

A complex interplay of genetic and environmental risk factors influence global brain structural alterations associated with brain health and disease. Epigenome-wide association studies (EWAS) of global brain imaging phenotypes have the potential to reveal the mechanisms of brain health and disease and can lead to better predictive analytics through the development of risk scores.

We perform an EWAS of global brain volumes in Generation Scotland using peripherally measured whole blood DNA methylation (DNAm) from two assessments, (i) at baseline recruitment, ~6 years prior to MRI assessment (N = 672) and (ii) concurrent with MRI assessment (N=565). Four CpGs at baseline were associated with global cerebral white matter, total grey matter, and whole-brain volume (Bonferroni p≤7.41×10^−8^, β_range_ = −1.46x10^−6^ to 9.59 × 10^−7^). These CpGs were annotated to genes implicated in brain-related traits, including psychiatric disorders, development, and ageing. We did not find significant associations in the meta-analysis of the EWAS of the two sets concurrent with imaging at the corrected level.

These findings reveal global brain structural changes associated with DNAm measured ~6 years previously, indicating a potential role of early DNAm modifications in brain structure. Although concurrent DNAm was not associated with global brain structure, the nominally significant findings identified here present a rationale for future investigation of associations between DNA methylation and structural brain phenotypes in larger population-based samples.

## Introduction

Global brain structure is influenced by genetic and environmental factors, and has previously been associated with health and disorder traits across the lifetime [1–3]. For instance, changes in global grey and white matter have been observed in a number of psychiatric and neurological disorders, including schizophrenia [4], major depressive disorder (MDD) [3], bipolar disorder [5], Rett syndrome [6], and Alzheimer’s disease [7]. Previous studies have also found age-related reductions in both grey and white matter [8,9].

Such global brain structural changes in both health and disease may reflect genetic and environmental factors and their impact. While previous studies have focussed on revealing the genetic architecture of brain structure, there are now opportunities to explore genetic and environmental risk factors through epigenetics, which correlate with changes in gene expression by modulating the genome in different cell types, without altering the underlying genome sequence [10]. One such process, DNA methylation (DNAm), implicates the covalent addition of a methyl group to a cytosine nucleotide followed by guanine in DNA, resulting in Cytosine-phosphate-Guanine (CpG) sites [10].

DNAm is modulated by both genetic and environmental factors, and may thus aid in identifying genetic and environmental contributions to health and disease [11]. Several brain- related traits and diseases are associated with variation in DNAm. MDD, a moderately heritable disorder, has been associated with differential methylation at several CpG sites, with a methylation risk score explaining 1.75% of the variance in the disorder [12]. Further, in an epigenome-wide association study (EWAS) using blood, CpG sites associated with depressive symptoms were annotated to genes involved in axonal guidance [13]. Schizophrenia has been associated with epigenetic variation at multiple loci that contribute to the polygenicity of the disorder [14,15]. Finally, growing evidence has shown that DNAm can act as a proxy for the biological age of multiple tissues across life [16]. These studies indicate that it may be possible, in future, to utilize DNAm modifications as biomarkers for brain-related healthy traits and diseases and to identify novel mechanisms contributing to these traits.

In recent years, increasing efforts have been made to identify epigenetic correlates of brain phenotypes, using both blood and brain tissue [17,18]. To maximize statistical power, previous studies have focused on candidate genes and candidate epigenetic markers in relation to specific brain regions of interest, such as subcortical volumes in the hippocampus and amygdala, as well as cortical thickness and volume in Freesurfer-derived brain regions [18], although consistency between study findings is modest. Recent advances in high- throughput array technologies that can identify DNAm levels at over 450 K and 850 K locations along the genome have enabled researchers to identify DNAm-brain associations using a hypothesis-free approach using EWAS [19]. DNAm modifications in relation to brain phenotypes have also been identified in patients as opposed to healthy individuals, including in the frontal cortex in schizophrenia [20,21], hippocampal volume in MDD [22], in the cerebral cortex in Alzheimer’s disease [23], and in the frontal cortex in Parkinson’s disease [24]. Structural brain measures may therefore function as endophenotypes that can be used to assess the association between epigenetic modifications and brain health and disease.

The pathogenesis of psychiatric and neurodegenerative disorders has been associated with a multitude of cortical and subcortical brain regions with inconsistent results across studies [3,25–27], potentially indicating a role for whole-brain abnormalities in these disorders. Peripheral DNAm alterations associated with clinically relevant global brain structure may therefore further our mechanistic understanding of brain anatomy in both health and disease, may help to identify modifiable risk factors and may form a basis for the development of more accurate predictive risk scores capturing a wider array of potential influences.

The majority of the studies mentioned above used whole blood as a surrogate tissue for the brain due to inaccessibility of the brain ante-mortem. Although DNAm is reported to be tissue- and cell type-specific, similarities between blood and brain DNAm have also been identified [28]. In addition, whole blood has successfully been used in the past to identify meaningful epigenetic differences in brain-related traits, as shown above [18].

Here, we sought to assess DNAm associations with Magnetic Resonance Imaging (MRI) global brain structural phenotypes, including cerebral white matter, total grey matter, and whole-brain volume using the Illumina Infinium MethylationEPIC array, capturing DNAm at approximately 850 K CpG sites [29]. Using DNAm measured ~6 years prior to MRI data collection, we examined whether CpG sites were associated with global brain structure at a later timepoint in N = 672 individuals. We then investigated whether concurrently measured DNAm was associated with global brain structure in N = 565 individuals.

## Methods

### Study population: Generation Scotland: Scottish Family Health Study (GS:SFHS)

GS:SFHS is a large, family-based epidemiological study aiming to investigate the genetics of health and disease in approximately 24,000 individuals aged 18–98 years across Scotland. Data collected between 2006 and 2011 consists of genetic, DNA methylation, and environmental variables [30,31]. GS:SFHS received ethical approval from NHS Tayside Research Ethics Committee (REC reference number 05/S1401/89) and has Research Tissue Bank Status (reference: 20/ES/0021). Written informed consent was obtained from all participants.

A total of N = 9,618 participants from GS responded when re-contacted at a later timepoint, and further data on mental health, specifically depression, was obtained. N = 1,188 were recruited for brain scanning, and approximately N = 700 with both DNAm and neuroimaging data were available at the time of the current study. Details of recruitment and study information have been reported previously [32,33]. The study was supported by the Wellcome Trust through a Strategic Award (reference 104036/Z/14/Z). Written consent at each stage of the study was obtained from all participants.

Two timepoints were used for the current study: blood samples were collected at baseline measurement (2006–2011), and concurrently with neuroimaging data (2015–2019).

## Phenotypes

### Global brain volumes

T1 images were processed using standard ENIGMA protocols [34] with FreeSurfer 5.3 and all output was visually quality checked. Manual edits were applied as required to correct for inclusion of skull tissue, exclusion of brain tissue or for errors in parcellation. Global measures were extracted from the final output following all edits. Manual editing, although necessary, did introduce a degree of subjective bias, therefore ‘editing’ was included as a binary covariate (values: yes/no). Further, as the complete set of T1s was processed, quality checked and edited in two parts, ‘batch’ was also included as a covariate.

We used 3 global volume measures in the current study. Total cerebral white matter includes hyperintensities and excludes anything that is not white matter. Total grey matter is rendered by the sum of the cortex within the left and right hemispheres, as well as subcortical and cerebellar grey matter. Finally, whole-brain volume includes both grey and white matter, and corresponds to brain volume without the brain stem, ventricles, cerebrospinal fluid, and choroid plexus.

### Baseline lifestyle factors and MDD status

Body mass index (BMI) was calculated using height (m) and weight (kg) as measured by clinical staff at baseline recruitment. Participants were asked to report the number of units of alcohol consumed during the past week and their smoking status (never, former, current); pack years was used to measure heaviness of smoking in current smokers by multiplying the number of cigarette packs (20 cigarettes/pack) smoked per day by the number of years a person has smoked [35]. MDD status was assessed at baseline using the Structured Clinical Interview of the Diagnostic and Statistical Manual, version IV (SCID) [36]. Participants with no MDD were defined as those individuals who did not fulfil criteria for a current or previous MDD diagnosis following the SCID interview.

### Concurrent lifestyle factors and MDD status

At the follow-up assessment, participants were sent study packages that included questionnaires. Here, BMI was calculated using height (m) and weight (kg). Participants also recorded the number of units consumed during the past week, whether they were current, former, or non-smokers, and (if they smoked) the number of cigarettes smoked in an average week. Finally, MDD status was ascertained through the Composite International Diagnostic Interview-Short Form (CIDI-SF) [37], and participants with no MDD were those individuals who did not fulfil criteria for current or previous MDD diagnoses based on responses.

## DNA methylation

Baseline DNAm data was pre-processed and quality-checked for all individuals by Amador et al. in 2019 [38]. At the concurrent timepoint, samples were placed on the array at two different time points and were therefore processed separately. The main difference between processing and analysis pipelines related to how key covariates were adjusted for. At baseline these were regressed out during pre-processing, whereas for the concurrent batches they were included as covariates in downstream analyses. However, across all batches, standard quality check (QC) and pre-processing steps with regards to sample and probe exclusions were identical (see below). We note however that differences in the processing resulted in different numbers of final CpG sites included for analysis.

Cross-reactive (N = 42,558) and polymorphic (N = 10,971) CpGs, obtained from McCartney et al. (2016) were removed from both the baseline and concurrent DNAm datasets [39] .

### Baseline DNA methylation

Genome-wide DNAm data profiled from whole blood samples was available for 9,873 individuals in GS:SFHS using the Illumina Human-MethylationEPIC BeadChip [29]. Samples were obtained and DNA was extracted between 2006–2011. DNAm profiling using the Illumina Human-MethylationEPIC BeadChip [29] was performed in two sets (in 2016, set A_N_ = 5101; in 2019, set B_N_ = 4,450) and pre-processing and QC was conducted once the second set was released, as detailed in Amador et al. [38,40,41]. Participants were removed due to a number of reasons, including sex mismatch (N_removed_ =24), having more than 1% CpG sites with a detection p-value>0.05 (N_removed_=52), being an outlier for bisulphite conversion control probes (N_removed_=1), having a median methylated signal intensity more than 3 standard deviations lower than expected (N_removed_=74), and other technical and dataset-specific issues (N_removed_=602, see Supplementary Materials). A total of 10,495 CpG sites were removed due to low beadcount, poor detection p-value, and sub-optimal binding.

R package ‘minfi’ was used to read in the IDAT files, compute M and beta values, and remove probes with large detection p-values, and to compute principal components (PC) of control probes. Correction was then applied for [1] technical variation, where M values were included as outcome variables in a mixed linear model adjusting for appointment date and Sentrix ID (random effects), jointly with Sentrix position, batch, clinic, year, weekday, and 10 PCs (fixed effects); and [2] biological variation by fitting residuals of [1] as outcome variables in a second mixed linear model adjusting for genetic and common family shared environmental contributions (random effects classed as G: common genetic; K: kinship; F: nuclear family; C: couple; and S: sibling) and sex, age, and estimated cell type proportions (CD8T, CD4T, NK, Bcell, Mono, Gran) (fixed effects) [42]. The final number of CpG sites that converged for these analyses was 674,246 across the 22 autosomes.

### Concurrent DNA methylation

Genome-wide DNAm data profiled from whole blood samples was available for a total of 710 individuals using the Illumina Human-MethylationEPIC BeadChip [29]. Pre-processing was carried out in two separate sets (N_set 1_=404; N_set 2_=306) intended as discovery and replication datasets, by Walker et al. [43,44]. Meffil [45] was use to remove samples if: there was a mismatch between self-reported and methylation-predicted sex and if >0.5% of probes failed the detection p- value threshold (>0.01); probes were removed if >1% samples failed the detection p- value >0.01 and if >5% of samples failed the beadcount threshold (N = 3). In addition, samples were removed if they showed evidence of dye bias and they were outliers for the bisulphite conversion control probes. ShinyMethyl [46] was then used to plot the log median intensity of methylated and unmethylated signals per array and inspect the output from the control probes; outlying samples detected by visual inspection were excluded. Meffil [45] was then used again to remove any additional samples who had a sex mismatch. PC plots were made using the first two methylation principal components and any additional outlying samples on the basis of these plots were removed. Finally, data were normalized using the dasen method in wateRmelon, and M-values were generated using the beta2m function in lumi [47]. The final number of CpG sites after pre-processing was N = 768,068 (set 1) and N = 765,695 (set 2) across the 22 autosomes.

## Statistical methods

### Epigenome-wide association

We used the ‘limma’ package [48] in R to run linear regression models for both baseline and concurrent DNAm data, where each CpG was included as an outcome variable. Brain cortical volumes, specifically cerebral white matter, total grey matter, and whole brain volume were included as predictor variables in separate EWAS at each DNAm timepoint. The R code for these analyses is available in the Supplementary Materials.

Covariates for each model using baseline DNAm were MRI site (to account for different data collection sites; see Supplementary Materials), age, age^2^, sex, intracranial volume, and set (to account for different DNAm data pre-processing sets). Due to the impact of lifestyle factors on DNAm [49–52], BMI, alcohol units, smoking status, and pack years were also included as covariates. Lastly, due to the increased prevalence of MDD in the dataset, MDD status was included as a covariate in all models. Technical (batch, appointment date) and biological (relatedness, cell type estimations, methylation principal components) variables were regressed out during pre-processing and were not included as covariates in downstream analyses. After QC, there were 674,246 CpGs and epigenome-wide significance was determined by a Bonferroni correction (0.05/674,246, p ≤ 7.41 × 10^−8^).

For both sets at the concurrent DNAm timepoint, covariates for each model were DNAm batch, 5 cell type proportion estimations (granulocytes, natural killer cells, B- lymphocytes, CD4 + T-lymphocytes and CD8 + T-lymphocytes), MRI site, age, age^2^, sex, intercranial volume, BMI, smoking status, number of cigarettes smoked/week, alcohol units, MDD status, and 20 methylation PCs. Bonferroni correction was applied based on the number of CpGs remaining in each set after QC (set 1: 0.05/768,068 CpGs, p ≤ 6.51x10^−8^; set 2: 0.05/765,695 CpGs, p ≤ 6.52x10^−8^).

The Blood Brain DNA Methylation Comparison Tool [53] (http://epigenetics.essex.ac.Uk/bloodbrain/) investigates the correlation between DNAm from whole blood and four brain regions (prefrontal cortex, entorhinal cortex, superior temporal gyrus, and cerebellum) for all probes on the Illumina 450 K array [54]. We used this resource to investigate the strength of correlation between the two tissues for CpGs identified here.

### Meta-analysis using METAL – concurrent timepoint

At the concurrent timepoint, in set 1, N = 331 individuals were available with global volume and methylation data after QC and N = 234 were available in set 2. Meta-analysis of these two datasets was performed in METAL [55] using p-value based analysis (N = 565). The meta-analysis was based on N = 769,263 CpGs across both sets and a Bonferroni correction (0.05/769,263) was used to define epigenome-wide significance (p ≤ 6.49x10^−8^).

### Pathway analysis

We annotated CpG sites to genes through the Infinium MethylationEPIC BeadChip database [29]. The database provides information about genes, chromosome location, start and end sites, and other features.

We used missMethyl [56], accessed via methylGSA [57], to assess pathway enrichment for differentially-methylated CpG sites. The package allows correction for biases in the representation of genes on the Infinium BeadChip. Gene Ontology (GO) terms were accessed using the msigdbr package [58]. Pathways included in the analysis were all GO pathways of size 1–250 genes inclusive. CpG sites included in the analysis were those significant at a threshold of p < 1x10^−5^, as used in previous studies [59]. Information on GO pathways can be accessed via www.geneontology.org using Gene Ontology identifiers, comprised of ‘GO’ followed by a string of numbers (e.g., GO:0000000).

### Power analysis – concurrent timepoint

Since the concurrent data was formed by two smaller samples of pre-processed data, we additionally conducted power analysis to determine whether our concurrent samples had sufficient power to detect a significant effect. This was conducted using effect sizes from the baseline data to inform the power calculations. We used the ‘pwr.f2.test’ function in package ‘pwr’ in R and the set parameters were as follows:

1. Regression coefficients: DNAm batch, 5 cell type estimations (granulocytes, natural killer cells, B-lymphocytes, CD4 + T-lymphocytes and CD8 + T-lymphocytes), MRI site, age, age^2^, sex, intercranial volume, BMI, smoking status, number of cigarettes smoked/week, alcohol units, MDD status, 20 methylation principal components.

2. Effect size: we input the largest effect size identified in EWAS at baseline (N = 672) for each global volume.

3. Significance level: to adjust for multiple testing correction (FDR), the p-value for a single potential test was set based on the number of CpG sites in each dataset (set 1: 0.05/768,068 = 6.51x10^−8^; set 2: 0.05/765,695 = 6.53x10^−8^).

4. Power: to observe different power percentages, we input 60%, 80%, 90%, 95% and 99% power.

## Results

### Demographic characteristics

There were N = 672 individuals in the baseline EWAS, N = 331 in the set 1 concurrent EWAS, and N = 234 in the set 2 concurrent EWAS. Demographic characteristics for all individuals are presented in Table 1. Further descriptive characteristics regarding global volumes are presented in Supplementary Table 1.

**Table 1.** Demographic characteristics for individuals with global volume data, including lifestyle variables and MDD. “-“ indicates that there was no data of the sort for the respective dataset. Former smokers at the baseline measurement were split into those who quit less than a year ago and those who quit more than a year ago; at the concurrent timepoint, this division is not made.

Demographic characteristics	Baseline (N = 672)	Concurrent set 1 (N = 331)	Concurrent set 2 (N = 234)
Age – Mean (SD), range	52.29 (9.93), 18–75	60.45 (8.42), 28–78	59.61 (10.21), 28–81
Sex			
Female	406	193	132
Male	266	138	102
Set			
1	621	-	-
2	51	-	-
BMI – Mean (SD), range	27.13 (4.96), 15.96–56.60	27.48 (5.18), 16.42–51.75	28.23 (5.31), 19–20-52.81
Alcohol units – Mean (SD), range	10.53 (16.44), 0–326	7.12 (8.91), 0–60	7.39 (9.67), 0–60
Smoking status			
Current smoker	83	16	12
Former smokers (quit < 1 year ago)	10	124	92
Former smokers (quit > 1 year ago)	208		
Never smoked tobacco	371	191	130
Pack years – Mean (SD), range	7.59 (14.56), 0–111	-	-
Cigarettes smoked/week			
1–10 cigarettes	-	10	6
11–20 cigarettes	-	10	9
MDD status			
Cases	121	83	83
Controls	551	248	151

### Baseline EWAS

**Figure 1.**
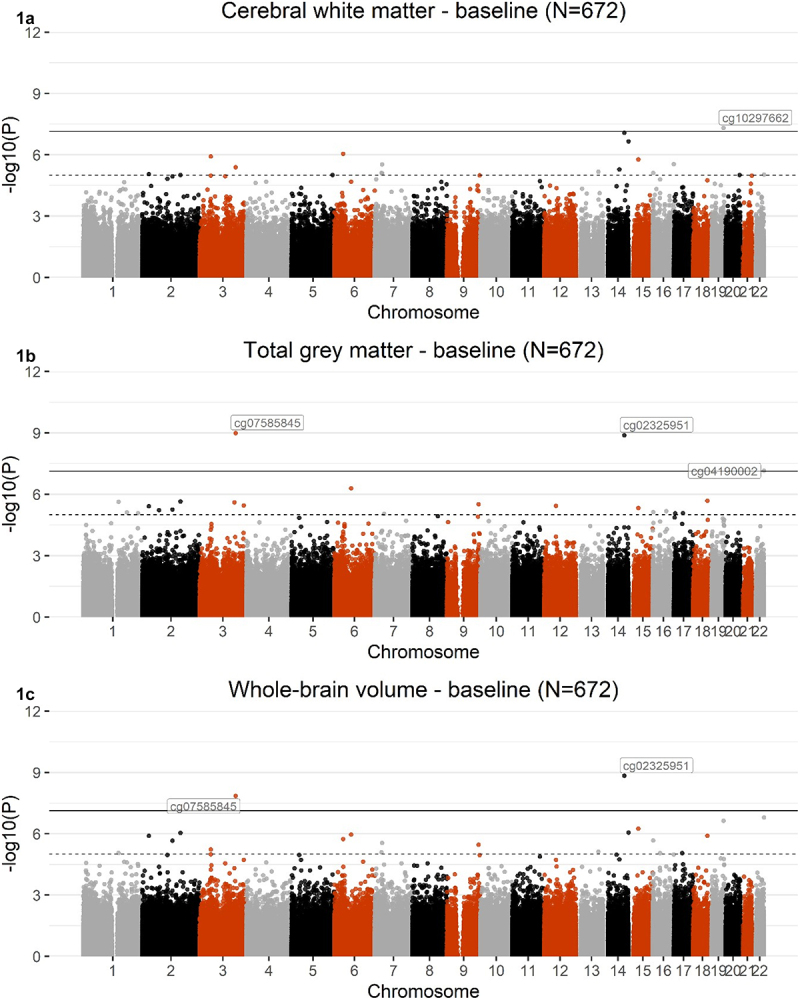
Manhattan plots showing the results from EWASs of cerebral white matter (1A), total grey matter (1B), and whole-brain volume (1 C), using baseline DNAm data (N = 672). The black line defines the threshold for epigenome-wide significance (p ≤ 7.41x10^−8^) and the dotted line defines CpG sites at p ≤ 1x10^−5^. Epigenome-wide significant hits for each phenotype are labelled on the graph.

Baseline EWAS identified 1, 3, and 2 CpG sites that were associated with cerebral white matter, total grey matter, and whole-brain volume, respectively (p ≤ 7.41x10^−8^). Both CpGs associated with whole brain volume were also associated with total grey matter and were significantly hypermethylated. One CpG site associated with cerebral white matter and one associated with total grey matter were hypomethylated. As shown in Figure 1a-c, CpG associations with grey matter were stronger than with white matter. Information about each CpG site is shown in Table 2.

**Table 2.** CpG sites significantly associated with cerebral white matter, total grey matter, and whole-brain volume (N = 672), along with gene annotations (Gene), chromosome (c), standardized effect size (β), nominal (P-value) and multiple comparison-corrected p-values (P-corr). Traits previously associated with each CpG site were extracted from EWAS catalogues (http://www.ewascatalog.org/, association between traits and CpGs on Illumina 450 K array at p ≤ 1.0x10^−4^; and http://www.bioapp.org/ewasdb/ [60],), association between traits and CpGs on Illumina 450 K and EPIC arrays at p ≤ 1.0x10^−3^). Gene information was extracted from the GWAS catalogue (https://www.ebi.ac.uk/gwas/; associations between traits and SNPs at p < 1.0x10^− 5^). All associations included in the table from these two catalogues are genome-wide significant.

Phenotype	CpG site	Gene	C	β	P-value	P-corr	CpG – previously associated traits	Gene – previously associated traits
Total grey matter	cg07585845 (EPIC)	-	3	9.59x10^−7^	1.02x10^−9^	0.0007	-	-
Whole- brain volume	cg07585845 (EPIC)	-	3	4.47x10^−7^	1.38x10^−8^	0.009		
Total grey matter	cg02325951 (450 K)	*FOXN3*	14	6.53x10^−7^	1.31x10^−9^	0.0009	Sex (p = 2x10^−54^; 1.8x10^−42^; [54])	Acute myeloid leukaemia (p = 8x10^−21^ p = 3x10^−14^; [61]) Heel bone mineral density (p = 2x10^−12^; [62]) Intelligence (p = 1x10^−11^; [79]) Self-reported educational attainment (p = 8x10^−11^; [80]) Cognitive function measurement (p = 2x10^−9^; [80]) Mathematical ability (p = 3x10^− 9^; [80]) Smoking status measurement (p = 7x10^−9^; [63]) Risk-taking behaviour (p = 8x10^− 9^; [64])
Whole- brain volume	cg02325951 (450 K)	*FOXN3*	14	3.26x10^−7^	1.45x10^−9^	0.001		
Cerebral white matter	cg10297662 (EPIC)	*PNKP*	19	−1.46x10^−6^	4.92x10^−8^	0.03	-	Involved in DNA repair; mutations at locus associated with microcephaly, seizures, and developmental delay [73]
Total grey matter	cg04190002 (450 K)	*SHANK3*	22	−3.75x10^−7^	7.31x10^−9^	0.04	Sex (p = 5.4x10^− 19^; [79])	Self-reported educational attainment (p = 2x10^−20^; [80]) Mathematical ability (p = 1x10^− 17^; [80]) Cognitive function measurement (p = 3x10^−12^; [80]) Schizophrenia (p = 3x10^−12^; [82])

### Correlation between whole blood DNAm and four brain regions

We used the Blood Brain DNA Methylation Comparison Tool [53] to investigate the correlation between blood and brain methylation measurements for two of the CpGs identified here, located on the 450 K array, and four brain regions. cg04190002 was strongly correlated with prefrontal cortex (r = 0.579, p = 6.55x10^−8^), entorhinal cortex (r = 0.564, p = 2.94x10^−7^), superior temporal gyrus (r = 0.598, p = 1.5x10^−8^), and cerebellum (r = 0.663, p = 3.02x10^−10^), while cg02325951 was strongly correlated with prefrontal cortex (r = 0.858, p = 1.73x10^−22^), entorhinal cortex (r = 0.868, p = 1.19x10^−22^), and superior temporal gyrus (r = 0.871, p = 3.32x10^−24^).

### Baseline pathway analysis

Enrichment of differentially methylated regions in biological pathways was analysed using missMethyl [56], where an over-representation analysis of GO pathways was performed for sets of genes annotated to CpG sites differentially expressed at p<1x10^−5^ (N _cerebral white matter_: 19, N _total grey matter_: 22, N _whole-brainvolume_: 21).

There were no over-represented pathways after multiple correction. A number of brain-related biological processes, molecular functions, and cellular components were included in the top 10 significant pathways (Supplementary Table 2). For instance, guanylate kinase-associated protein clustering, which facilitates assembly of post-synaptic density of neurons (GO:0097117), was found to be over-represented for all three imaging phenotypes (cerebral white matter nominal p-value = 0.0007; total grey matter nominal p-value = 0.001; whole-brain volume nominal p-value = 0.0009). Positive regulation of synapse structural plasticity (GO:0051835) was over-represented in both cerebral white matter (nominal p-value = 0.002) and total grey matter (nominal p-value = 0.002). Finally, forebrain generation of neurons (GO:0021872; nominal p-value = 0.001) was over-represented for cerebral white matter.

### Concurrent EWAS

**Figure 2.**
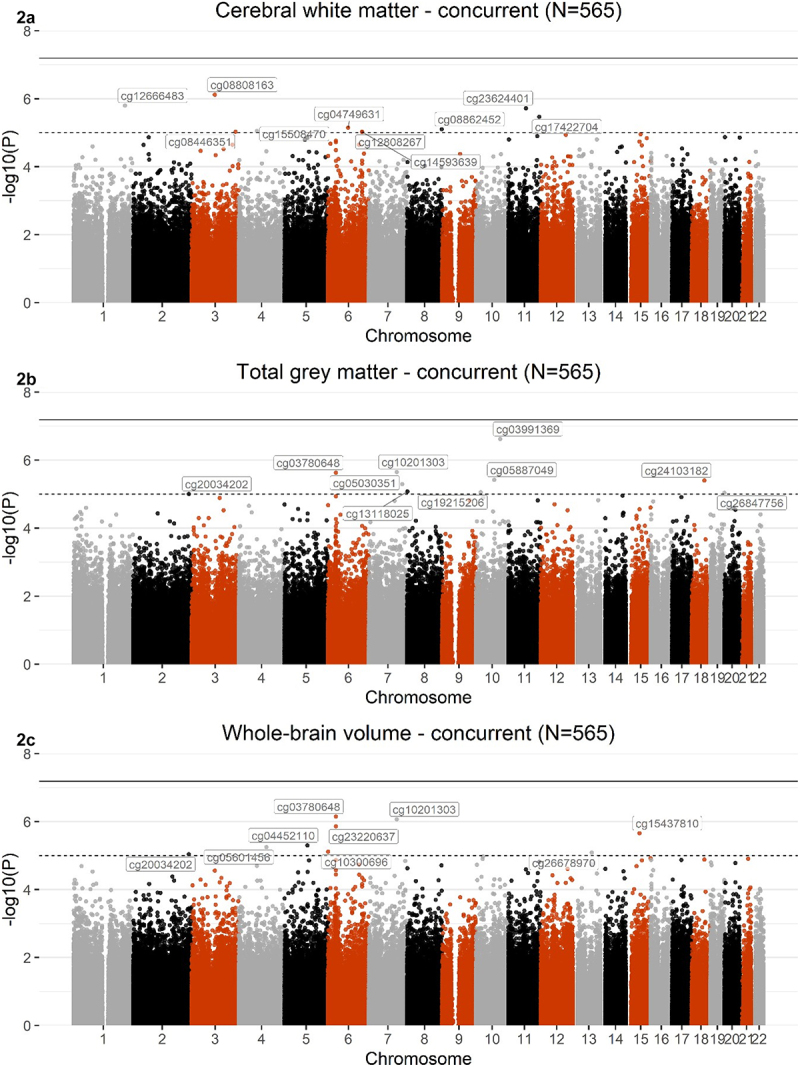
Manhattan plots showing meta-analysis of EWAS of cerebral white matter (2A), total grey matter (2B), and whole-brain volume (2 C), across the 2 concurrent sets ^(N^set 1^=331; N^set 2^=234; N^total^=565)^. The black line defines the threshold for epigenome-wide significance (p ≤ 6.5x10^−8^) and the dotted line defines p ≤ 1x10^−5^. CpGs that met a significance of p ≤ 1x10^−5^ are labelled on the graph.

Meta-analysis of EWAS across the two concurrent sets did not reveal any Bonferroni-corrected CpG sites associated with any of the global volumes (Figure 2a-c). A list of the top 10 CpGs associated with cerebral white matter (EWAS_set 1_ β_range_=4.71x10−6- 6.53x10^−6^; EWAS_set 2_β_range_=1.02x10^−5^−8.75x10^−6^) total grey matter (EWAS^set 1^ β^range^=6.71x10^−6^−8.03x10^−6^; EWAS^set 2^β_range_=1.03x10^−5^−8.84x10^−6^), and whole-brain volume (EWAS^set 1^β^range^=2.69x10−6−4.05x10−6; EWAS_set 2_ β_range_= 6.23x10^−6^ −6.69x10^−6^), is presented in Supplementary Tables 3–5. Genes annotated to these top 10 CpGs have previously been implicated in brain-related phenotypes, including psychiatric disorders (MDD [65–68], schizophrenia [69]), neurodegenerative disorders (neurofibrillary tangles and PHF-tau measurement in Alzheimer’s Disease [70]), and cognitive traits (mathematical ability, self- reported educational attainment [71]). Results reported here are nominal and should be supported by further large-scale cohorts.

### Concurrent pathway analysis

As above, enrichment of differentially methylated regions in specific pathways was assessed using missMethyl [50] for sets of genes annotated to CpG sites differentially expressed at p<1x10−5 (N_cerebral white matter_: 10, N_total grey matter_: 10, N_whole-brainvolume_: 9). There were no over-represented pathways following FDR adjustment for multiple comparisons. The top 10 most significant pathways for each phenotype indicated a pattern of phenotype-specific biological processes, molecular functions, and cellular components (Supplementary Table 6). For instance, over-represented pathways in cerebral white matter included myelination (GO:0042552; nominal p-value = 0.002), ensheathment of neurons (GO:0007272; nominal p-value = 0.002), axon ensheathment (GO:0008366; nominal p-value = 0.001), glial cell development (GO:0021782; nominal p-value = 0.001) and glial cell differentiation (GO:0010001; nominal p-value = 0.004). Total grey matter over-represented pathways included glutamate catabolic process to aspartate (GO:0019550; nominal p-value = 0.0009) and to 2-oxoglutarate (GO:0019551; nominal p-value = 0.0009). Finally, over-represented pathways in whole-brain volume included several MHC-related biological processes, including regulation (GO:0002586; nominal p-value = 0.001) and negative regulation (GO:0002587; nominal p-value = 0.0009) of antigen processing and presentation of peptide antigen via MHC class II, negative regulation of antigen processing and presentation of peptide or polysaccharide antigen via MHC class II (GO:0002581; nominal p-value = 0.001), as well as N- acetyllactosaminide beta-1,3-N-acetylglucosaminyltransferase (GO:0008532, molecular function, nominal p-value = 0.001), an enzyme encoded by the gene *B3GNT2*, which is highly expressed in whole-brain, hippocampus, amygdala, cerebellum, and caudate nucleus (https://www.uniprot.org/uniprot/Q9Z222).

### Power curves for concurrent data

**Figure 3.**
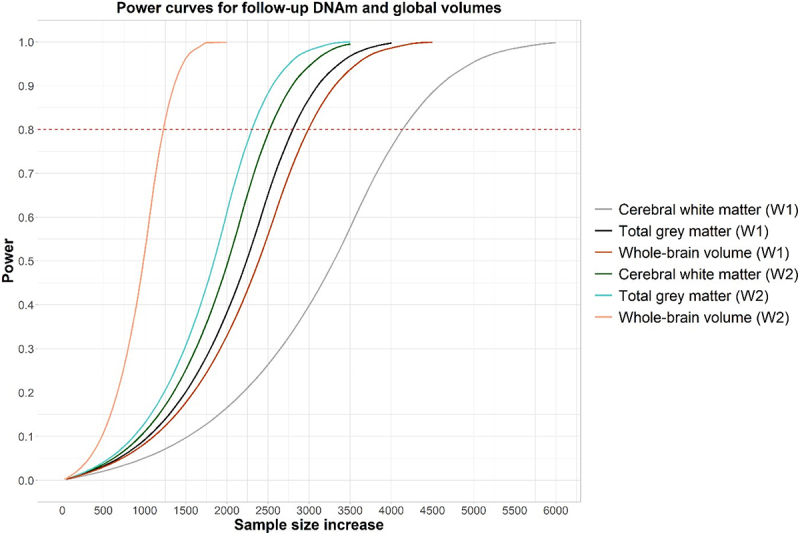
Power curves for cerebral white matter, total grey matter, and whole-brain volume calculated separately for set 1 and set 2. The x-axis indicates how many participants would be needed to detect an effect with 60%, 80%, 90%, 95% or 99% power at p < 6.51x10^−8^ (set 1 (W1)) and p < 6.53x10^−8^ (set 2 (W2)) with 36 regression coefficients included in the linear model. Effect sizes were calculated based on the largest effect size obtained in EWAS for each phenotype at baseline.

Power curves for the three imaging phenotypes are presented in Figure 3. Further details, including effect size for each phenotype, are included in Supplementary Tables 7 and 8. These indicate that approximately 1,000–6,000 individuals (depending on phenotype) would be needed to detect an effect after multiple correction.

## Discussion

We report a number of significant associations between DNAm measured ~6 years prior to MRI data collection and cerebral white matter (N_significant CpGs_=1), total grey matter (N_significant CpGs_=3), and whole-brain volume (N_significant CpGs_=2) (N=672), annotated to genes involved in brain-related traits. There were no significant associations between DNAm collected concurrently with MRI data (N = 565). In addition, pathway analysis did not uncover any significant findings for either the baseline or concurrent analyses. Power analysis of the concurrent data using baseline data for effect size confirmed that approximately 1,000–6,000 individuals (depending on phenotype) would be needed to detect a statistically significant effect.

For the analysis of associations between DNAm measured at baseline and cortical volumes ~6 years later, one CpG associated with cerebral white matter, cg10297662, was annotated to *PNKP*. This CpG site has not previously been associated with any other traits, to the best of our knowledge. *PNKP* is involved in DNA repair following ionizing radiation or oxidative damage [72] and is expressed in a number of tissues, including the brain. Mutations in this gene have been associated with a number of neural conditions, including microcephaly, developmental delay, seizures, and cerebellar ataxia [73,74]. These mutations have been shown to lead to white matter defects, which is the phenotype investigated here [75]. Previous evidence also indicates that loss of *PNKP* strongly impacts oligodendrocytes, leading to white matter abnormalities [76]. Efforts should be made to identify whether the relationship between *PNKP* mutations and defects in white matter is mediated by differential DNAm at specific sites.

Two CpGs, cg07585845 and cg02325951, were associated with both total grey matter and whole-brain volume. cg07585845 has not been previously associated with any traits nor annotated to any genes. cg02325951 was previously associated with sex in a study investigating methylation trajectories across human foetal brain development (p = 2x10^−54^ [77];). The gene to which cg02325951 is annotated, *FOXN3*, is involved in several physiological processes, such as development, ageing, obesity, and cancer and is expressed in multiple tissues, including the forebrain and midbrain. Further, animal studies show that mutations within the gene have been associated with craniofacial defects [78]. In addition, *FOXN3* has previously been associated with several brain-related phenotypes in previous GWAS, including intelligence (p = 1x10^−11^ [79];), self-reported educational attainment (p = 8x10^−11^), cognitive function measurement (p = 2x10^−9^), and mathematical ability (p = 3x10^−9^) [80]. These cognition-related phenotypes have previously been associated with whole brain volume, where higher cognition was associated with a larger brain size [72]. Future studies should investigate whether DNAm localized to *FOXN3* plays a role in cognition development through modifications in whole-brain volume.

Finally, in addition to the two CpGs above, total grey matter was also associated with cg04190002, a CpG previously associated with sex in newborns (p = 5.4x10^−19^ [81];). The CpG is annotated to *SHANK3*, which encodes multidomain scaffold proteins of the postsynaptic density connecting neurotransmitter receptors, among other membrane proteins and is expressed in the cerebral cortex and the cerebellum. The gene has previously been associated with a host of brain disorders and traits, including self- reported educational attainment (p = 2x10^−20^), mathematical ability (p = 1x10^−17^), cognitive function measurement (p = 3x10^−12^) [80] and schizophrenia (p = 3x10^−9^ [82];), and mutations have previously been associated with autism spectrum disorder [83]. These disorders in turn have been associated with changes in grey matter [84], and future studies should investigate whether these psychiatric disorders are also associated with differential DNAm at cg04190002, and other probes localized to *SHANK3*, as well as explore whether associations are mediated by global brain phenotypes.

Blood and brain methylation measures for both cg02325951 and cg04190002 (both CpGs on the 450 K array) were strongly correlated, indicating that whole blood is a suitable proxy tissue for investigating associations with brain phenotypes, at least for these probes. Future studies exploring DNAm in relation to global brain phenotypes and associated traits may therefore benefit from whole blood DNAm measurements.

DNAm profiled at a different timepoint to phenotype measurement has previously yielded interesting results. Barbu et al. (2020) found that a methylation risk score calculated from DNAm profiled 4–11 years prior to MDD diagnosis was significantly associated with incident cases who were well at DNAm measurement but went on to develop MDD [12]. Clark et al. (2020) similarly associated DNAm profiled in MDD patients at baseline with MDD status 6 years later [85]. These previous findings indicate that DNAm measured prior to phenotype measurement may provide meaningful insight into phenotype development and change across time. The findings above relating DNAm measured previously to MRI scans may therefore aid in the investigation of epigenetic differences in brain-related disease and health at a later timepoint, although further longitudinal replication is needed to verify these associations.

Associations between DNAm measured concurrently to MRI scans did not yield any significant findings. Power calculations using the baseline data to derive effect size showed that approximately 1,000–6,000 participants (depending on phenotype) would be needed to uncover a significant effect at epigenome-wide level. This number is supported by previous studies, such as Jia et al. (2019), who analysed 3,337 individuals across 11 cohorts as part of ENIGMA to find 2 CpGs significantly associated with hippocampal volume [19]. This may indicate that null findings were due to lack of power at the concurrent timepoint. Null findings here should serve as a stimulus for larger collaborations and meta-analyses in future.

Further, effect sizes for both timepoints were much smaller than those identified in previous studies that analysed larger sample sizes in specific brain regions [19] (largest baseline effect size: 1.46x10^−6^; largest concurrent effect size: 1.06 × 10^−6^), which suggests that findings here should be interpreted with caution. The results here indicate that global associations with DNAm may be weaker than those at a regional level. Future studies may therefore benefit from focussing on lobe- and region-specific correlates of DNAm.

At the concurrent timepoint, DNAm data was pre-processed and quality-checked in 2 sets, resulting in a different number of final CpGs (N_CpG set 1_=768,068; N_CpG set 2_=765,695). Pearson’s correlations between the EWAS betas from set 1 and set 2 across all CpGs were *r* = 0.02 (95% C.I. = 0–0.102), *r* = 0.04 (95% C.I. = 0–122), and *r* = 0.03 (95% C.I. = 0–0.112) for cerebral white matter, total grey matter, and whole brain volume, respectively. When restricting CpGs to those with a nominal p-value (≤0.05), the beta correlations were slightly higher, although not strong: *r* = 0.17 (95% C.I. = 0.089–0.249), *r* = 0.18 (95% C.I. = 0.099–0.259), and *r* = 0.22 (95% C.I. = 0.14–0.297) for cerebral white matter, total grey matter, and whole-brain volume, respectively. The low effect size correlations may be a further reflection of the small sample investigated here.

There are limitations to the current study. Firstly, we report DNAm changes in whole blood, which may not be representative of brain phenotypes. However, two of the CpGs identified here, located on the 450 K array, were strongly correlated with DNAm in four brain regions [53]. Although previous studies have shown that there is considerable agreement between blood and brain [28], future studies should explore DNAm changes in the brain in post-mortem samples where possible to uncover biological mechanisms underpinning brain structure within the same tissue. Further, findings at baseline may indicate that some DNAm changes lie upstream of brain structural changes, although effect sizes for each CpG were small compared to previous concurrent EWAS of brain regions [18,19]. In addition, we cannot test the direction of association between brain structural changes and DNAm. In future, studies may apply Mendelian Randomization to investigate whether DNAm may be on the causal path to brain structure alterations in brain health and disease. Finally, in the current study we focussed on global brain phenotypes to explore whether global brain-related changes, previously associated with psychiatric and neurological disorders, are associated with DNAm alterations. Previous evidence includes DNAm associations at both global and regional level [18], and it may be that DNAm may provide more insight into region-specific alterations in relation to brain health and disease.

In conclusion, we report an EWAS of global cortical brain volumes using DNAm data collected ~6 years prior to MRI data collection in 672 individuals and an EWAS meta-analysis of cortical brain volumes using DNAm measured concurrently to MRI data in 565 individuals, both part of a large, population-based cohort. Using baseline DNAm data, we find four CpGs significantly associated with cortical brain volumes ~6 years later, all of which are annotated to genes implicated in brain-related phenotypes. We did not find significant associations at the concurrent timepoint. Findings here should be interpreted with caution, and future studies should aim to determine further links between DNAm changes and brain structure and function, to highlight our understanding of this relationship in health and disease

## Supplementary Material

Supplemental Material
