# Supplementary material for: Epigenome-wide association study of global cortical volumes in generation Scotland: Scottish family health study
Source: Epigenetics. 2021 Nov 23;17(10):1143–58. doi: 10.1080/15592294.2021.1997404 (PMC9542280; doi:10.1080/15592294.2021.1997404)
Supplement: Supplemental Material [file KEPI_A_1997404_SM1570.docx]

**Supplementary Materials**

**Sample removal for baseline DNAm data**

In addition to sample and probe removal at baseline, 602 further participants were removed due to technical and cohort-specific reasons. These included being a genetic outlier (N=7), being outliers on the MDS (N=7) and QC (N=55) outlier plot, being male outliers (samples that did not cluster with other male samples, despite being recorded as male, N=40), unreliable self-report data for other phenotypes (N=3), having a possible XXY chromosome (N=1), providing saliva DNA instead of blood (N=10), being duplicate samples (N=95), technical failure (N=14) and being a concurrent sample but not a baseline sample (N=373).

**R code**

1. **Epigenome-wide association study:** EWAS was performed through a locally created pipeline; the phenotype input file should contain a column of participant IDs and separate columns for each phenotype; the pipeline also takes as input a covariate file, which should contain a column of participant IDs and separate columns with each covariate. The script below is the main part of the pipeline, indicating the regression model for cerebral white matter EWAS; EWAS for all other traits both at baseline and follow-up, were performed in a similar fashion.

Input sample: 762 (initial input file contains 762 individuals)

Complete phenotype and covariates: 672 (by merging with DNA methylation data and covariates, there are 672 individuals remaining for EWAS)

Probes excluded: 0 (no probes were excluded for this analysis)

Model: ~global.cerebral.wm + mri_site + set + agesq + units + bmi + ever_smoke + pack_years + dep_status

Design matrix: ~ (Intercept) + global.cerebral.wm + mri_site + set3 + agesq + units + bmi + ever_smoke + pack_years + dep_status

EWAS sample size: 672 (final EWAS sample size)

EWAS probes: 674246 (final number of EWAS probes)

1. **METAL**: meta-analysis was carried out through METAL using a command line tool on Linux. The following commands were provided to METAL (this is for cerebral white matter in concurrent DNAm set 1; METAL for all other traits were performed in a similar fashion).

SEPARATOR WHITESPACE (column separator in input files)

MARKER ID (column containing CpGs to be analysed)

PVALUE P.Value (EWAS p-value column)

EFFECT EFFECT (EWAS effect size column)

WEIGHTLABEL N (number of participants in each EWAS)

PROCESS set1cwmformetal.txt (process set 1 EWAS results)

PROCESS set2cwmformetal.txt (process set 2 EWAS results)

ANALYZE (run METAL to analyse the 2 EWAS)

1. **Power analysis**: power analysis was run using the “pwr” package in R:

pwr.f2.test(u = number of covariates in linear model, f2 = effect size, sig.level = p-value, power = power percentage)

1. **Pathway analysis**: pathway analysis was conducted using missMethyl; the following is for CpGs at 1x10^-5^; pathway analysis for all other traits was performed in a similar fashion:

cwm_sign_pathways=gometh(

sig.cpg=sign_cwm, # CpGs at 1x10^-5^

all.cpg = all_cwm, # all CpGs in analysis

collection = c("GO", "KEGG"), # Search in Gene Ontology and KEGG pathways

array.type = c("EPIC"), # type of DNAm array

plot.bias = FALSE,

prior.prob = TRUE,

anno = NULL,

equiv.cpg = TRUE,

fract.counts = TRUE)

**MRI scanning parameters**

In Aberdeen, participants were imaged on a 3T Philips Achieva TX-series MRI system (Philips Healthcare, Best, Netherlands) with a 32 channel phased-array head coil and a back facing mirror (software version 5.1.7; gradients with maximum amplitude 80 mT/m and maximum slew rate 100 T/m/s). A projector and “Presentation” (Neurobehavioural Systems Inc, Berkeley, CA, USA) version 18.1 were used for the presentation of task-based fMRI.

In Dundee, participants were scanned using a Siemens 3T Prisma-FIT (Siemens, Erlangen, Germany) with 20 channel head and neck phased array coil and a back facing mirror (Syngo E11, gradient with max amplitude 80 mT/m and maximum slew rate 200 T/m/s). A magnetic resonance compatible LCD screen was used to display fMRI (NordicNeuroLab, Bergen, Norway) task stimuli using “Presentation” version 20.0.

| **EWAS timepoint** | **Mean (SD)** |
| --- | --- |
| Cerebral white matter (baseline) | 455691.8 (55333.65) |
| Cerebral white matter (set 1) | 457255.7 (55438.9) |
| Cerebral white matter (set 2) | 459672.4 (58244.91) |
| Total grey matter (baseline) | 581342.7 (58054.28) |
| Total grey matter (set 1) | 562963.7 (54614.58) |
| Total grey matter (set 2) | 599443.9 (56385.34) |
| Whole-brain volume (baseline) | 1068678 (105962.7) |
| Whole-brain volume (set 1) | 1050634 (104946.9) |
| Whole-brain volume (set 2) | 1091711 (108445.7) |

**Supplementary Table 1.** Descriptive statistics of cerebral white matter, total grey matter, and whole-brain volume at baseline (N=672) and concurrent timepoints (N=565).

| **Brain phenotype** | **Ontology** | **Term** | **N** | **DE** | **P-value DE** | **FDR** |
| --- | --- | --- | --- | --- | --- | --- |
| **Cerebral white matter** | BP | guanylate kinase-associated protein clustering | 1 | 1 | 0.0007 | 1 |
|  | BP | nucleotide-excision repair | 1 | 1 | 0.0009 | 1 |
|  | BP | DNA excision | 1 | 1 | 0.0009 | 1 |
|  | MF | polynucleotide 3'-phosphatase activity | 1 | 1 | 0.0009 | 1 |
|  | BP | DNA 3' dephosphorylation | 1 | 1 | 0.0009 | 1 |
|  | BP | DNA 3' dephosphorylation involved in DNA repair | 1 | 1 | 0.0009 | 1 |
|  | BP | polynucleotide 3' dephosphorylation | 1 | 1 | 0.0009 | 1 |
|  | BP | forebrain generation of neurons | 63 | 2 | 0.001 | 1 |
|  | BP | melanin biosynthetic process from tyrosine | 2 | 1 | 0.001 | 1 |
|  | BP | positive regulation of synapse structural plasticity | 2 | 1 | 0.002 | 1 |
| **Total grey matter** | MF | COPII adaptor activity | 1 | 1 | 0.0009 | 1 |
|  | BP | guanylate kinase-associated protein clustering | 1 | 1 | 0.001 | 1 |
|  | BP | nuclear mRNA surveillance of mRNA 3'-end processing | 2 | 1 | 0.002 | 1 |
|  | BP | nuclear retention of pre-mRNA with aberrant 3'-ends at the site of transcription | 2 | 1 | 0.002 | 1 |
|  | BP | positive regulation of fever generation by positive regulation of prostaglandin secretion | 2 | 1 | 0.002 | 1 |
|  | BP | positive regulation of ERK1 and ERK2 cascade via TNFSF11-mediated signaling | 2 | 1 | 0.002 | 1 |
|  | MF | endothelial differentiation G protein-coupled receptor binding | 2 | 1 | 0.002 | 1 |
|  | MF | Edg-2 lysophosphatidic acid receptor binding | 2 | 1 | 0.002 | 1 |
|  | BP | positive regulation of synapse structural plasticity | 2 | 1 | 0.002 | 1 |
|  | BP | circadian temperature homeostasis | 3 | 1 | 0.003 | 1 |
| **Whole-brain volume** | BP | nucleotide-excision repair | 1 | 1 | 0.0009 | 1 |
|  | BP | DNA excision | 1 | 1 | 0.0009 | 1 |
|  | MF | polynucleotide 3'-phosphatase activity | 1 | 1 | 0.0009 | 1 |
|  | BP | DNA 3' dephosphorylation | 1 | 1 | 0.0009 | 1 |
|  | BP | DNA 3' dephosphorylation involved in DNA repair | 1 | 1 | 0.0009 | 1 |
|  | BP | polynucleotide 3' dephosphorylation | 1 | 1 | 0.0009 | 1 |
|  | BP | guanylate kinase-associated protein clustering | 1 | 1 | 0.0009 | 1 |
|  | MF | vitamin-K-epoxide reductase (warfarin-insensitive) activity | 1 | 1 | 0.001 | 1 |
|  | BP | melanin biosynthetic process from tyrosine | 2 | 1 | 0.001 | 1 |
|  | BP | positive regulation of fever generation by positive regulation of prostaglandin secretion | 2 | 1 | 0.002 | 1 |

**Supplementary Table 2.** Pathway analysis - enrichment of top 10 differentially methylated genes in EWAS of cerebral white matter, total grey matter, and whole-brain volume at baseline. BP=biological process; MF=molecular function; CC=cellular component. N=number of genes participating in pathway; DE=number of genes that are differentially methylated; P-value DE=p-value for over-representation of the pathway. FDR=false discovery rate.

| **CpG site** | **Gene** | **N** | **Z-score** | **P-value** | **Direction** | **CpG look-up** | **Gene look-up** |
| --- | --- | --- | --- | --- | --- | --- | --- |
| cg08808163 (EPIC) | MINA | 564 | -4.944 | 7.61x10^-7^ | -- |  | May play a role in cell proliferation regulation (<https://www.genecards.org/cgi-bin/carddisp.pl?gene=RIOX2>) |
| cg12666483 (EPIC) | CENPF | 564 | 4.799 | 1.59x10^-6^ | ++ |  | Glomerular filtration rate (p=2x10^-15^; (1))  Birth weight (p=5x10^-10^; (2))  YLK40 (inflammatory biomarker) measurement (p=6x10^-9^; (3))  Neurofibrillary tangles measurement (p=6x10^-8^; (4)) |
| cg23624401 (450K) | UCP2 | 564 | -4.763 | 1.91 x10^-6^ | -- | HIV infection (p=1.9x10^-7^; (5)) | May play a role in non-shivering thermogenesis, obesity, and diabetes (<https://www.genecards.org/cgi-bin/carddisp.pl?gene=UCP2>) |
| cg17422704 (450K) | - | 564 | -4.644 | 3.42 x10^-6^ | -- | HIV infection (p=3.1x10^-5^; (5)) | - |
| cg04749631 (450K) | PGM3; RWDD2A | 564 | -4.492 | 7.07 x10^-6^ | -- | Arm tissue fat (p=3.5x10^-4^; (6)) | PGM3: mediates glycogen formation and utilization; may play a role in diabetic nephropathy and neuropathy (<https://www.genecards.org/cgi-bin/carddisp.pl?gene=PGM3&keywords=PGM3>)  RWDD2A: platelet-derived growth factor BB measurement (p=3x10^-8^; (7)) |
| cg08862452 (450K) | PUF60 | 564 | 4.468 | 7.91 x10^-6^ | ++ | Gestational age (p=2.4x10^-8^; (8)) | Diastolic blood pressure (p=4x10^-8^; (9)) |
| cg15508470 (EPIC) | - | 564 | -4.442 | 8.9 x10^-6^ | -- |  | - |
| cg12808267 (EPIC) | ADGRG6 | 564 | -4.432 | 9.32 x10^-6^ | -- |  | Lung function (FEV/FEC ratio) (p=8x10^-227^; (10))  Body height (p=3x10^-55^; (10))  Body fat distribution (p=3x10^-33^; (11))  White matter microstructure (MD average across all tracts) (p=2x10^-8^; (12)) |
| cg08446351 (EPIC) | EIF2B5 | 564 | 4.432 | 9.35 x10^-6^ | ++ |  | Mutations associated with leukoencephalopathy with vanishing white matter (13,14) |
| cg14593639 (EPIC) | ADGRG6 | 564 | -4.430 | 9.41 x10^-6^ | -- |  | Lung function (FEV/FEC ratio) (p=8x10^-227^; (10))  Body height (p=3x10^-55^; (10))  Body fat distribution (p=3x10^-33^; (11))  White matter microstructure (MD average across all tracts) (p=2x10^-8^; (12)) |

**Supplementary Table 3.** Top 10 CpG sites associated with cerebral white matter using concurrent DNAm data (N=565), along with gene annotations, chromosome, standardised effect size, nominal and multiple comparison-corrected p-values. Background information for each CpG site was extracted from EWAS catalogues (<http://www.ewascatalog.org/>, association between traits and CpGs on Illumina 450K array at p≤1.0x10^-4)^; and <http://www.bioapp.org/ewasdb/> (EWASdb, (15), association between traits and CpGs on Illumina 450K and EPIC arrays at p≤1.0x10^-3^). Gene information was extracted from GWAS catalogue (<https://www.ebi.ac.uk/gwas/>; associations between traits and SNPs at p < 1.0x10^-5^). Direction: indicates direction of effect in EWAS of set 1 (first sign) and set 2 (second sign).

| **CpG site** | **Gene** | **N** | **Z-score** | **P-value** | **Direction** | **CpG look-up** | **Gene look-up** |
| --- | --- | --- | --- | --- | --- | --- | --- |
| cg03991369 (450K) | GOT1 | 565 | 5.167 | 2.38 x10^-7^ | ++ | Fetal vs adult liver (p=3.1x10^-35^;(16))  Rheumatoid arthritis (p=1.6x10^-10^; (17)) | AST levels (test for liver damage) (p=9x10^-10^; (18))  Adolescent idiopathic scoliosis (p=8x10^-8^; (19))  Important regulator of levels of glutamate, major excitatory neurotransmitter of the vertebrate CNS (<https://www.genecards.org/cgi-bin/carddisp.pl?gene=GOT1>) |
| cg10201303 (EPIC) | - | 565 | -4.733 | 2.22 x10^-6^ | -- |  | - |
| cg03780648 (450K) | HLA-DOA (MHC) | 565 | -4.723 | 2.32 x10^-6^ | -- | Age 4 vs age 0 (p=4.7x10^-75^; (20)) | Chronic hepatitis B virus infection (p=1x10^-23^; (21))  Neurofibrillary tangles measurement (p=5x10^-11^; (4))  Schizophrenia, ASD (p=1x10^-8^; (22)) |
| cg05887049 (EPIC) | ADK | 565 | -4.623 | 3.79 x10^-6^ | -- |  | Tooth eruption (p=6x10^-18^; (23))  Platelet count (p=4x10^-17^; (24))  BMI (p=1x10^-12^; (25)) |
| cg24103182 (EPIC) | TCF4 | 565 | 4.613 | 3.97 x10^-6^ | ++ |  | Unipolar depression (p=2x10^-27^; (26))  Mathematical ability (p=5x10^-23^; (27))  Self-reported educational attainment (p=9x10^-20^; (27))  Loneliness (p=4x10^-17^; (28))  Depressed affect (p=4x10^-17^; (29)) |
| cg05030351 (450K) | ATP6V0A4 | 565 | -4.561 | 5.09 x10^-6^ | -- | Phenylalanine (p=8.1x10^-3^; (6)) | Sleep duration (p=4x10^-10^; (30)) |
| cg13118025 (EPIC) | DLGAP2 | 565 | 4.455 | 8.38 x10^-6^ | ++ |  | Product of gene may play a role in synapse organisation and signalling in neuronal cells (<https://www.genecards.org/cgi-bin/carddisp.pl?gene=DLGAP2&keywords=DLGAP2>)  Neuroticism (p=6x10^-9^; (29))  PHF-tau measurement (p=8x10^-9^; (4))  Self-reported educational attainment (p=2x10^-8^; (27))  Feeling miserable (p=2x10^-8^; (31))  Depressive symptoms (p=4x10^-8^; (32)) |
| cg19215206 (EPIC) | MALRD1 | 565 | 4.445 | 8.81 x10^-6^ | ++ |  | Pulse pressure measurement (p=4x10^-16^; (33))  PHF-tau measurement (p=3x10^-9^; (4)) |
| cg26847756 (450K) | ZNF552 | 565 | 4.436 | 9.18 x10^-6^ | ++ | Rheumatoid arthritis (p=6.1x10^-8^; (17)) | May be involved in transcriptional regulation (<https://www.genecards.org/cgi-bin/carddisp.pl?gene=ZNF552&keywords=ZNF552>) |
| cg20034202 (450K) | B3GNT7 | 565 | -4.420 | 9.85 x10^-6^ | -- | Rheumatoid arthritis (p=2x10^-10^; (17)) | Resting heart rate (p=1x10^-17^; (34))  Monocyte count (p=3x10^-10^; (24)) |

**Supplementary Table 4.** Top 10 CpG sites associated with total grey matter using concurrent DNAm data (N=565). along with gene annotations, chromosome, standardised effect size, nominal and multiple comparison-corrected p-values. Background information for each CpG site was extracted from EWAS catalogues (<http://www.ewascatalog.org/>, association between traits and CpGs on Illumina 450K array at p≤1.0x10^-4)^; and <http://www.bioapp.org/ewasdb/> (EWASdb, (15), association between traits and CpGs on Illumina 450K and EPIC arrays at p≤1.0x10^-3^). Gene information was extracted from GWAS catalogue (<https://www.ebi.ac.uk/gwas/>; associations between traits and SNPs at p < 1.0x10^-5^). Direction: indicates direction of effect in EWAS of set 1 (first sign) and set 2 (second sign).

| **CpG site** | **Gene** | **N** | **Z-score** | **P-value** | **Direction** | **CpG look-up** | **Gene look-up** |
| --- | --- | --- | --- | --- | --- | --- | --- |
| cg03780648 (450K) | HLA-DOA (MHC) | 560 | -4.960 | 7.07x10^-7^ | -- | Age 4 vs age 0 (p=4.7x10^-75^; (20)) | Chronic hepatitis B virus infection (p=1x10^-23^; (21))  Neurofibrillary tangles measurement (p=5x10^-11^; (4))  Schizophrenia, ASD (p=1x10^-8^; (22)) |
| cg10201303 (EPIC) | - | 560 | -4.922 | 8.56 x10^-7^ | -- |  |  |
| cg23220637 (450K) | HLA-DOA (MHC) | 560 | -4.829 | 1.37 x10^-6^ | -- | Gestational age (p=2.3x10^-3^; (35)) | Chronic hepatitis B virus infection (p=1x10^-23^; (21))  Neurofibrillary tangles measurement (p=5x10^-11^; (4))  Schizophrenia, ASD (p=1x10^-8^; (22) |
| cg15437810 (EPIC) | PRTG | 560 | 4.735 | 2.19 x10^-6^ | ++ |  | Self-reported educational attainment (p=1x1^-11^; (27))  Risk-taking behaviour (p=3x10^-8^; (36))  Encoded protein associated with development of neurogenesis (<https://www.genecards.org/cgi-bin/carddisp.pl?gene=PRTG&keywords=PRTG>) |
| cg04452110 (450K) | ELL2 | 331 | -4.565 | 4.99 x10^-6^ | -? | Cholesterol esters to total lipids ratio in medium HDL (p=8.3x10^-4^; (6)) | Mean corpuscular haemoglobin concentration (p=5x10^-16^; (24))  Non-albumin protein levels (p=7x10^-16^; (37)) |
| cg05601456 (450K) | ARSJ | 560 | 4.538 | 5.67 x10^-6^ | ++ | Maternal underweight (p=2.7x10^-4^; (38)) | Body height (p=1x10^-15^; (39)) |
| cg10300696 (EPIC) | LOC285768 | 560 | -4.474 | 7.66 x10^-6^ | -- |  | - |
| cg26678970 (450K) | - | 560 | 4.461 | 8.17 x10^-6^ | ++ | Current vs never smoking (p=8.9x10^-5^; (40)) | - |
| cg20034202 (450K) | B3GNT7 | 560 | -4.436 | 9.19 x10^-6^ | -- | Rheumatoid arthritis (p=2x10^-10^; (17)) | Resting heart rate (p=1x10^-17^; (34))  Monocyte count (p=3x10^-10^; (24)) |
| cg14001239 (450K) | SVIL | 560 | 4.405 | 1.06 x10^-6^ | ++ | Fetal vs adult liver (p=1.1x10^-45^; (16))  Gestational age (p=3x10^-10^; (8))  Maternal smoking in pregnancy (p=6.4x10^-8^; (41)) | Glomerular filtration rate (p=3x10^-10^; (1))  PHF-tau measurement (p=6x10^-9^; (4))  DNA methylation (p=8x10^-9^; (42)) |

**Supplementary Table 5.** Top 10 CpG sites associated with whole-brain volume using concurrent DNAm data (N=565). along with gene annotations, chromosome, standardised effect size, nominal and multiple comparison-corrected p-values. Background information for each CpG site was extracted from EWAS catalogues (<http://www.ewascatalog.org/>, association between traits and CpGs on Illumina 450K array at p≤1.0x10^-4)^; and <http://www.bioapp.org/ewasdb/> (EWASdb, (15), association between traits and CpGs on Illumina 450K and EPIC arrays at p≤1.0x10^-3^). Gene information was extracted from GWAS catalogue (<https://www.ebi.ac.uk/gwas/>; associations between traits and SNPs at p < 1.0x10^-5^). Direction: indicates direction of effect in EWAS of set 1 (first sign) and set 2 (second sign).

| **Brain volume** | **Ontology** | **Term** | **N** | **DE** | **P-value DE** | **FDR** |
| --- | --- | --- | --- | --- | --- | --- |
| **Cerebral white matter** | BP | glial cell development | 114 | 2 | 0.001 | 1 |
|  | MF | oxidative phosphorylation uncoupler activity | 3 | 1 | 0.001 | 1 |
|  | BP | myelination | 129 | 2 | 0.002 | 1 |
|  | BP | ensheathment of neurons | 131 | 2 | 0.002 | 1 |
|  | BP | axon ensheathment | 131 | 2 | 0.002 | 1 |
|  | CC | eukaryotic translation initiation factor 2B complex | 6 | 1 | 0.003 | 1 |
|  | BP | glial cell differentiation | 215 | 2 | 0.004 | 1 |
|  | CC | ciliary transition fiber | 9 | 1 | 0.005 | 1 |
|  | BP | response to temperature stimulus | 226 | 2 | 0.005 | 1 |
|  | BP | heart trabecula formation | 13 | 1 | 0.006 | 1 |
| **Total grey matter** | MF | L-cysteine:2-oxoglutarate aminotransferase activity | 1 | 1 | 0.0004 | 1 |
|  | BP | response to transition metal nanoparticle | 2 | 1 | 0.0007 | 1 |
|  | BP | cellular carbohydrate biosynthetic process | 83 | 2 | 0.0009 | 1 |
|  | MF | phosphatidylserine decarboxylase activity | 2 | 1 | 0.0009 | 1 |
|  | BP | glutamate catabolic process to aspartate | 2 | 1 | 0.0009 | 1 |
|  | BP | glutamate catabolic process to 2-oxoglutarate | 2 | 1 | 0.0009 | 1 |
|  | MF | L-aspartate:2-oxoglutarate aminotransferase activity | 3 | 1 | 0.001 | 1 |
|  | BP | aspartate biosynthetic process | 3 | 1 | 0.001 | 1 |
|  | BP | fumarate metabolic process | 3 | 1 | 0.001 | 1 |
|  | MF | adenosine kinase activity | 1 | 1 | 0.001 | 1 |
| **Whole-brain volume** | BP | negative regulation of antigen processing and presentation of peptide antigen via MHC class II | 2 | 1 | 0.0009 | 1 |
|  | BP | negative regulation of antigen processing and presentation of peptide antigen | 4 | 1 | 0.0009 | 1 |
|  | BP | regulation of antigen processing and presentation of peptide antigen via MHC class II | 4 | 1 | 0.001 | 1 |
|  | BP | regulation of antigen processing and presentation of peptide antigen | 6 | 1 | 0.001 | 1 |
|  | MF | N-acetyllactosaminide beta-1 3-N-acetylglucosaminyltransferase activity | 8 | 1 | 0.001 | 1 |
|  | BP | negative regulation of antigen processing and presentation of peptide or polysaccharide antigen via MHC class II | 3 | 1 | 0.001 | 1 |
|  | BP | negative regulation of antigen processing and presentation | 8 | 1 | 0.002 | 1 |
|  | BP | regulation of antigen processing and presentation of peptide or polysaccharide antigen via MHC class II | 5 | 1 | 0.002 | 1 |
|  | BP | poly-N-acetyllactosamine biosynthetic process | 9 | 1 | 0.002 | 1 |
|  | BP | poly-N-acetyllactosamine metabolic process | 10 | 1 | 0.002 | 1 |

**Supplementary Table 6.** Pathway analysis - enrichment of top 10 differentially methylated genes in EWAS meta-analysis of cerebral white matter, total grey matter, and whole-brain volume at the concurrent timepoint. BP=biological process; MF=molecular function; CC=cellular component. N=number of genes participating in pathway; DE=number of genes that are differentially methylated; P-value DE=p-value for over-representation of the pathway. FDR=false discovery rate.

| **Global volume** | **Sample size** | **Effect size** | **P-value** | **Power** |
| --- | --- | --- | --- | --- |
| Cerebral white matter | 3558 | 0.01940985 | 6.51x10^-8^ | 0.6 |
| Total grey matter | 2412 | 0.02854804 | 6.51x10^-8^ | 0.6 |
| Whole-brain volume | 2573 | 0.02677562 | 6.51x10^-8^ | 0.6 |
| Cerebral white matter | 4132 | 0.01940985 | 6.51x10^-8^ | 0.8 |
| Total grey matter | 2799 | 0.02854804 | 6.51x10^-8^ | 0.8 |
| Whole-brain volume | 2987 | 0.02677562 | 6.51x10^-8^ | 0.8 |
| Cerebral white matter | 4586 | 0.01940985 | 6.51x10^-8^ | 0.9 |
| Total grey matter | 3105 | 0.02854804 | 6.51x10^-8^ | 0.9 |
| Whole-brain volume | 3313 | 0.02677562 | 6.51x10^-8^ | 0.9 |
| Cerebral white matter | 4976 | 0.01940985 | 6.51x10^-8^ | 0.95 |
| Total grey matter | 3368 | 0.02854804 | 6.51x10^-8^ | 0.95 |
| Whole-brain volume | 3594 | 0.02677562 | 6.51x10^-8^ | 0.95 |
| Cerebral white matter | 5746 | 0.01940985 | 6.51x10^-8^ | 0.99 |
| Total grey matter | 3886 | 0.02854804 | 6.51x10^-8^ | 0.99 |
| Whole-brain volume | 4148 | 0.02677562 | 6.51x10^-8^ | 0.99 |

**Supplementary Table 7.** Power analysis results for set 1. Sample size: indicates how many participants are needed to detect a statistically significant effect with 60%, 80%, 90%, 95% or 99% power at p<6.51x10^-8^ (set 1 p-value) with 36 regression coefficients included in the linear model. Effect sizes were calculated based on the maximum effect size obtained in EWAS for each phenotype at baseline.

| **Global volume** | **Sample size** | **Effect size** | **P-value** | **Power** |
| --- | --- | --- | --- | --- |
| Cerebral white matter | 2171 | 0.03168751 | 6.53x10^-8^ | 0.6 |
| Total grey matter | 1985 | 0.03462878 | 6.53x10^-8^ | 0.6 |
| Whole-brain volume | 1060 | 0.06426812 | 6.53x10^-8^ | 0.6 |
| Cerebral white matter | 2518 | 0.03168751 | 6.53x10^-8^ | 0.8 |
| Total grey matter | 2302 | 0.03462878 | 6.53x10^-8^ | 0.8 |
| Whole-brain volume | 1226 | 0.06426812 | 6.53x10^-8^ | 0.8 |
| Cerebral white matter | 2793 | 0.03168751 | 6.53x10^-8^ | 0.9 |
| Total grey matter | 2552 | 0.03462878 | 6.53x10^-8^ | 0.9 |
| Whole-brain volume | 1357 | 0.06426812 | 6.53x10^-8^ | 0.9 |
| Cerebral white matter | 3029 | 0.03168751 | 6.53x10^-8^ | 0.95 |
| Total grey matter | 2768 | 0.03462878 | 6.53x10^-8^ | 0.95 |
| Whole-brain volume | 1469 | 0.06426812 | 6.53x10^-8^ | 0.95 |
| Cerebral white matter | 3495 | 0.03168751 | 6.53x10^-8^ | 0.99 |
| Total grey matter | 3192 | 0.03462878 | 6.53x10^-8^ | 0.99 |
| Whole-brain volume | 1691 | 0.06426812 | 6.53x10^-8^ | 0.99 |

**Supplementary Table 8.** Power analysis results for set 2. Sample size: indicates how many participants are needed to detect a statistically significant effect with 60%, 80%, 90%, 95% or 99% power at p<6.53x10^-8^ (set 2 p-value) with 36 regression coefficients included in the linear model. Effect sizes were calculated based on the maximum effect size obtained in EWAS for each phenotype at baseline.

**References**

1. Wuttke M, Li Y, Li M, Sieber KB, Feitosa MF, Gorski M, et al. A catalog of genetic loci associated with kidney function from analyses of a million individuals. Nat Genet [Internet]. 2019 Jun 1 [cited 2020 Nov 10];51(6):957–72. Available from: /pmc/articles/PMC6698888/?report=abstract

2. Plotnikov D, Williams C, Guggenheim JA. Association between birth weight and refractive error in adulthood: A Mendelian randomisation study. Br J Ophthalmol [Internet]. 2020 Feb 1 [cited 2020 Nov 10];104(2):214–9. Available from: https://pubmed.ncbi.nlm.nih.gov/31097437/

3. Zhang R, Song J, Isgren A, Jakobsson J, Blennow K, Sellgren CM, et al. Genome-wide study of immune biomarkers in cerebrospinal fluid and serum from patients with bipolar disorder and controls. Transl Psychiatry [Internet]. 2020 Dec 1 [cited 2020 Nov 10];10(1). Available from: /pmc/articles/PMC7026056/?report=abstract

4. Wang H, Yang J, Schneider JA, De Jager PL, Bennett DA, Zhang HY. Genome-wide interaction analysis of pathological hallmarks in Alzheimer’s disease. Neurobiol Aging [Internet]. 2020 Sep 1 [cited 2020 Nov 10];93:61–8. Available from: https://pubmed.ncbi.nlm.nih.gov/32450446/

5. Gross AM, Jaeger PA, Kreisberg JF, Licon K, Jepsen KL, Khosroheidari M, et al. Methylome-wide Analysis of Chronic HIV Infection Reveals Five-Year Increase in Biological Age and Epigenetic Targeting of HLA. Mol Cell [Internet]. 2016 Apr 21 [cited 2020 Nov 10];62(2):157–68. Available from: /pmc/articles/PMC4995115/?report=abstract

6. Battram T, Richmond RC, Baglietto L, Haycock PC, Perduca V, Bojesen SE, et al. Appraising the causal relevance of DNA methylation for risk of lung cancer. Int J Epidemiol [Internet]. 2019 Oct 1 [cited 2020 Nov 10];48(5):1493–504. Available from: https://academic.oup.com/ije/article/48/5/1493/5573019

7. Ahola-Olli A V., Würtz P, Havulinna AS, Aalto K, Pitkänen N, Lehtimäki T, et al. Genome-wide Association Study Identifies 27 Loci Influencing Concentrations of Circulating Cytokines and Growth Factors. Am J Hum Genet [Internet]. 2017 Jan 5 [cited 2020 Nov 10];100(1):40–50. Available from: /pmc/articles/PMC5223028/?report=abstract

8. Spiers H, Hannon E, Schalkwyk LC, Smith R, Wong CCY, O’Donovan MC, et al. Methylomic trajectories across human fetal brain development. Genome Res [Internet]. 2015 Mar 1 [cited 2020 Nov 9];25(3):338–52. Available from: http://www.genome.org/cgi/doi/10.1101/gr.180273.114.

9. Warren HR, Evangelou E, Cabrera CP, Gao H, Ren M, Mifsud B, et al. Genome-wide association analysis identifies novel blood pressure loci and offers biological insights into cardiovascular risk. Nat Genet [Internet]. 2017 Mar 1 [cited 2020 Nov 10];49(3):403–15. Available from: /pmc/articles/PMC5972004/?report=abstract

10. Kichaev G, Bhatia G, Loh PR, Gazal S, Burch K, Freund MK, et al. Leveraging Polygenic Functional Enrichment to Improve GWAS Power. Am J Hum Genet [Internet]. 2019 Jan 3 [cited 2020 Nov 9];104(1):65–75. Available from: https://pubmed.ncbi.nlm.nih.gov/30595370/

11. Rask-Andersen M, Karlsson T, Ek WE, Johansson Å. Genome-wide association study of body fat distribution identifies adiposity loci and sex-specific genetic effects. Nat Commun [Internet]. 2019 Dec 1 [cited 2020 Nov 10];10(1). Available from: /pmc/articles/PMC6341104/?report=abstract

12. Zhao B, Zhang J, Ibrahim JG, Luo T, Santelli RC, Li Y, et al. Large-scale GWAS reveals genetic architecture of brain white matter microstructure and genetic overlap with cognitive and mental health traits (n = 17,706). Mol Psychiatry [Internet]. 2019; Available from: http://dx.doi.org/10.1038/s41380-019-0569-z

13. Fogli A, Wong K, Eymard-Pierre E, Wenger J, Bouffard J-P, Goldin E, et al. Cree leukoencephalopathy and CACH/VWM disease are allelic at theEIF2B5 locus. Ann Neurol [Internet]. 2002 Oct 1 [cited 2020 Nov 10];52(4):506–10. Available from: http://doi.wiley.com/10.1002/ana.10339

14. Dietrich J, Lacagnina M, Gass D, Richfield E, Mayer-Pröschel M, Noble M, et al. EIF2B5 mutations compromise GFAP+ astrocyte generation in vanishing white matter leukodystrophy. Nat Med [Internet]. 2005 Mar 20 [cited 2020 Nov 10];11(3):277–83. Available from: http://www.nature.com/naturemedicine

15. Liu D, Zhao L, Wang Z, Zhou X, Fan X, Li Y, et al. EWASdb: Epigenome-wide association study database. Nucleic Acids Res [Internet]. 2019 Jan 8 [cited 2020 Nov 9];47(D1):D989–93. Available from: /pmc/articles/PMC6323898/?report=abstract

16. Bonder MJ, Kasela S, Kals M, Tamm R, Lokk K, Barragan I, et al. Genetic and epigenetic regulation of gene expression in fetal and adult human livers. BMC Genomics [Internet]. 2014 Oct 4 [cited 2020 Nov 10];15(1). Available from: https://pubmed.ncbi.nlm.nih.gov/25282492/

17. Liu Y, Aryee MJ, Padyukov L, Fallin MD, Hesselberg E, Runarsson A, et al. Epigenome-wide association data implicate DNA methylation as an intermediary of genetic risk in rheumatoid arthritis. Nat Biotechnol [Internet]. 2013 Feb [cited 2020 Nov 10];31(2):142–7. Available from: https://pubmed.ncbi.nlm.nih.gov/23334450/

18. Moon S, Kim YJ, Han S, Hwang MY, Shin DM, Park MY, et al. The Korea Biobank Array: Design and Identification of Coding Variants Associated with Blood Biochemical Traits. Sci Rep [Internet]. 2019 Dec 1 [cited 2020 Nov 10];9(1). Available from: https://pubmed.ncbi.nlm.nih.gov/30718733/

19. Liu J, Zhou Y, Liu S, Song X, Yang XZ, Fan Y, et al. The coexistence of copy number variations (CNVs) and single nucleotide polymorphisms (SNPs) at a locus can result in distorted calculations of the significance in associating SNPs to disease. Hum Genet [Internet]. 2018 Jul 1 [cited 2020 Nov 10];137(6–7):553–67. Available from: https://pubmed.ncbi.nlm.nih.gov/30019117/

20. Xu CJ, Bonder MJ, Söderhäll C, Bustamante M, Baïz N, Gehring U, et al. The emerging landscape of dynamic DNA methylation in early childhood. BMC Genomics [Internet]. 2017 Jan 5 [cited 2020 Nov 10];18(1):25. Available from: http://bmcgenomics.biomedcentral.com/articles/10.1186/s12864-016-3452-1

21. Jiang DK, Ma XP, Yu H, Cao G, Ding DL, Chen H, et al. Genetic Variants in Five Novel Loci Including CFB and CD40 Predispose to Chronic Hepatitis B. Hepatology [Internet]. 2015 Jul 1 [cited 2020 Nov 10];62(1):118–28. Available from: https://pubmed.ncbi.nlm.nih.gov/25802187/

22. Meta-analysis of GWAS of over 16,000 individuals with autism spectrum disorder highlights a novel locus at 10q24.32 and a significant overlap with schizophrenia. Mol Autism [Internet]. 2017 [cited 2020 Nov 10];8:21. Available from: https://pubmed.ncbi.nlm.nih.gov/28540026/

23. Geller F, Feenstra B, Zhang H, Shaffer JR, Hansen T, Esserlind AL, et al. Genome-wide association study identifies four loci associated with eruption of permanent teeth. PLoS Genet [Internet]. 2011 Sep [cited 2020 Nov 10];7(9). Available from: https://pubmed.ncbi.nlm.nih.gov/21931568/

24. Chen MH, Raffield LM, Mousas A, Sakaue S, Huffman JE, Moscati A, et al. Trans-ethnic and Ancestry-Specific Blood-Cell Genetics in 746,667 Individuals from 5 Global Populations. Cell [Internet]. 2020 Sep 3 [cited 2020 Nov 10];182(5):1198-1213.e14. Available from: https://pubmed.ncbi.nlm.nih.gov/32888493/

25. Pulit SL, Stoneman C, Morris AP, Wood AR, Glastonbury CA, Tyrrell J, et al. Meta-Analysis of genome-wide association studies for body fat distribution in 694 649 individuals of European ancestry. Hum Mol Genet [Internet]. 2019 Jan 1 [cited 2020 Nov 10];28(1):166–74. Available from: https://pubmed.ncbi.nlm.nih.gov/30239722/

26. Howard DM, Adams MJ, Clarke T-K, Hafferty JD, Gibson J, Shirali M, et al. Genome-wide meta-analysis of depression identifies 102 independent variants and highlights the importance of the prefrontal brain regions. bioRxiv. 2018.

27. Lee JJ, Wedow R, Okbay A, Kong E, Maghzian O, Zacher M, et al. Gene discovery and polygenic prediction from a genome-wide association study of educational attainment in 1.1 million individuals. Nat Genet. 2018;50(8):1112–21.

28. Day FR, Ong KK, Perry JRB. Elucidating the genetic basis of social interaction and isolation. Nat Commun [Internet]. 2018 Dec 1 [cited 2020 Nov 10];9(1). Available from: https://pubmed.ncbi.nlm.nih.gov/29970889/

29. Nagel M, Jansen PR, Stringer S, Watanabe K, De Leeuw CA, Bryois J, et al. Meta-analysis of genome-wide association studies for neuroticism in 449,484 individuals identifies novel genetic loci and pathways. Nat Genet [Internet]. 2018 Jul 1 [cited 2020 Nov 10];50(7):920–7. Available from: https://pubmed.ncbi.nlm.nih.gov/29942085/

30. Noordam R, Bos MM, Wang H, Winkler TW, Bentley AR, Kilpeläinen TO, et al. Multi-ancestry sleep-by-SNP interaction analysis in 126,926 individuals reveals lipid loci stratified by sleep duration. Nat Commun [Internet]. 2019 Dec 1 [cited 2020 Nov 10];10(1). Available from: https://pubmed.ncbi.nlm.nih.gov/31719535/

31. Nagel M, Watanabe K, Stringer S, Posthuma D, Van Der Sluis S. Item-level analyses reveal genetic heterogeneity in neuroticism. Nat Commun [Internet]. 2018 Dec 1 [cited 2020 Nov 10];9(1). Available from: https://pubmed.ncbi.nlm.nih.gov/29500382/

32. Baselmans BML, Jansen R, Ip HF, van Dongen J, Abdellaoui A, van de Weijer MP, et al. Multivariate genome-wide analyses of the well-being spectrum. Nat Genet. 2019 Mar 1;51(3):445–51.

33. Evangelou E, Warren HR, Mosen-Ansorena D, Mifsud B, Pazoki R, Gao H, et al. Genetic analysis of over 1 million people identifies 535 new loci associated with blood pressure traits. Nat Genet [Internet]. 2018 Oct 1 [cited 2020 Nov 10];50(10):1412–25. Available from: https://pubmed.ncbi.nlm.nih.gov/30224653/

34. Eppinga RN, Hagemeijer Y, Burgess S, Hinds DA, Stefansson K, Gudbjartsson DF, et al. Identification of genomic loci associated with resting heart rate and shared genetic predictors with all-cause mortality. Nat Genet [Internet]. 2016 Dec 1 [cited 2020 Nov 10];48(12):1557–63. Available from: https://pubmed.ncbi.nlm.nih.gov/27798624/

35. Bohlin J, Håberg SE, Magnus P, Reese SE, Gjessing HK, Magnus MC, et al. Prediction of gestational age based on genome-wide differentially methylated regions. Genome Biol. 2016 Oct 7;17(1).

36. Karlsson Linnér R, Biroli P, Kong E, Meddens SFW, Wedow R, Fontana MA, et al. Genome-wide association analyses of risk tolerance and risky behaviors in over 1 million individuals identify hundreds of loci and shared genetic influences. Nat Genet [Internet]. 2019 Feb 1 [cited 2020 Nov 9];51(2):245–57. Available from: /pmc/articles/PMC6713272/?report=abstract

37. Kanai M, Akiyama M, Takahashi A, Matoba N, Momozawa Y, Ikeda M, et al. Genetic analysis of quantitative traits in the Japanese population links cell types to complex human diseases. Nat Genet [Internet]. 2018 Mar 1 [cited 2020 Nov 10];50(3):390–400. Available from: https://pubmed.ncbi.nlm.nih.gov/29403010/

38. Sharp GC, Lawlor DA, Richmond RC, Fraser A, Simpkin A, Suderman M, et al. Maternal pre-pregnancy BMI and gestational weight gain, offspring DNA methylation and later offspring adiposity: Findings from the Avon Longitudinal Study of Parents and Children. Int J Epidemiol [Internet]. 2015 Aug 1 [cited 2020 Nov 10];44(4):1288–304. Available from: https://pubmed.ncbi.nlm.nih.gov/25855720/

39. Kichaev G, Bhatia G, Loh PR, Gazal S, Burch K, Freund MK, et al. Leveraging Polygenic Functional Enrichment to Improve GWAS Power. Am J Hum Genet [Internet]. 2019 Jan 3 [cited 2020 Sep 17];104(1):65–75. Available from: https://pubmed.ncbi.nlm.nih.gov/30595370/

40. Joehanes R, Just AC, Marioni RE, Pilling LC, Reynolds LM, Mandaviya PR, et al. Epigenetic Signatures of Cigarette Smoking. Circ Cardiovasc Genet. 2016;436–47.

41. Joubert BR, Håberg SE, Nilsen RM, Wang X, Vollset SE, Murphy SK, et al. 450K epigenome-wide scan identifies differential DNA methylation in newborns related to maternal smoking during pregnancy. Environ Health Perspect. 2012 Oct;120(10):1425–31.

42. Zhang Q, Marioni RE, Robinson MR, Higham J, Sproul D, Wray NR, et al. Genotype effects contribute to variation in longitudinal methylome patterns in older people. Genome Med [Internet]. 2018 Oct 22 [cited 2020 Nov 10];10(1). Available from: https://pubmed.ncbi.nlm.nih.gov/30348214/
